# Application of Atomic Force Microscopy as Advanced Asphalt Testing Technology: A Comprehensive Review

**DOI:** 10.3390/polym14142851

**Published:** 2022-07-13

**Authors:** Qijian Ouyang, Zhiwei Xie, Jinhai Liu, Minghui Gong, Huayang Yu

**Affiliations:** 1 School of Civil Engineering and Transportation, South China University of Technology, Guangzhou 510641, China; ctouyangqj@mail.scut.edu.cn (Q.O.); 201930095207@mail.scut.edu.cn (Z.X.); 201930093197@mail.scut.edu.cn (J.L.); 2 State Key Laboratory of High Performance Civil Engineering Materials, Nanjing 210096, China; gongminghui@cnjsjk.cn; 3 Guangdong Provincial Key Laboratory of Modern Civil Engineering Technology, Guangzhou 510641, China

**Keywords:** AFM, asphalt binder, asphalt materials, microstructure of asphalt, macro performance of asphalt

## Abstract

In the past three decades, researchers have engaged in the relationship between the composition, macro performance, and microstructure of asphalt. There are many research results in the use of atomic force microscopy (AFM) to study the microstructure and related mechanisms of asphalt. Based on previous studies, the performance of asphalt from its microstructure has been observed and analyzed, and different evaluation indices and modification methods have been proposed, providing guidance toward improving the performance of asphalt materials and benefiting potential applications. This review focuses on the typical application and analysis of AFM in the study of the aging regeneration and modification properties of asphalt. Additionally, this review introduces the history of the rheological and chemical testing of asphalt materials and the history of using AFM to investigate asphalt. Furthermore, this review introduces the basic principles of various modes of application of AFM in the microstructure of asphalt, providing a research direction for the further popularization and application of AFM in asphalt or other materials in the future. This review aims to provide a reference and direction for researchers to further popularize the application of AFM in asphalt and standardize the testing methods of AFM. This paper is also helpful in further exploring the relationship between the microstructure and macro performance of asphalt.

## 1. Introduction

AFM has been widely used in materials science [1,2,3], electrochemistry [4,5,6], life science [7,8,9], and other fields. With the advancement in AFM technology, the improvement in observation resolution, and the expansion of application scope, more quantitative analysis of the observed images has begun to be carried out. For example, in the field of biomedicine, the focus of most experimental studies has become the relationship between the structure and related functions of biological macromolecules, especially nucleic acids and proteins [10,11]. In materials science, AFM can not only obtain the information of the three-dimensional morphology and surface roughness of the material surface, but also obtain the difference in the distribution of the physical properties of the material surface such as impedance distribution [12,13] and dielectric constant [14].

In the field of road engineering, asphalt is a widely used building material, and the use of microstructure observation instruments such as AFM to observe asphalt has been the research direction of many experts and scholars in recent years. Given that many scholars have used AFM to study asphalt materials from various perspectives in the last two decades, there is a lack of reviews on the application of AFM in asphalt materials to help researchers quickly understand the progress in current research.

Second, in the field of the microstructural observation of asphalt using AFM and other microscopes, there is no authoritative specification to standardize the whole sample preparation and observation process. This review will allow the reader to become familiar with the progress and difficulties in the existing research and provide future researchers with a direction to further explore the changes in the asphalt microstructure. It also provides a research direction to improve existing testing methods, explore new research methods and ways, and verify the feasibility of other related research.

## 2. Chemical Tests of Asphalt Materials

Varied tests, indices, and specifications related to asphalt properties have been formulated and improved in recent years including chemical detection methods and the application of AFM. Conventional asphalt testing methods have proposed indices to reflect the performances of asphalt such as viscosity, properties in low and high temperature, etc. However, the direct relationship between the microstructure and macroscopic properties cannot be obtained by conventional asphalt rheological testing methods, leading to difficulties in the identification and quality control of asphalt products. Chemical detection methods allow for a better understanding of the link between the component, morphology, and properties of asphalt from a microscopic perspective. Furthermore, the application of some chemical detection methods with AFM provides a way to observe the microstructure and mechanical properties of asphalt. In this part, the chemical tests of asphalt are summarized and compared with AFM tests.

### 2.1. Chemical Detection Method

After years of development, the traditional testing methods for asphalt have become systematic, and so the performance of asphalt can be assessed in a more comprehensive manner [15,16,17]. While traditional experimental methods can represent the properties of asphalt at a macroscopic level, chemical analysis methods are increasingly being used in the testing of asphalt and asphalt mixtures in order to better understand the link between the composition, structure, and properties of asphalt mixture components from a microscopic perspective [18]. In recent years, common chemical analysis methods include Fourier transform infrared spectroscopy (FTIR), gel permeation chromatography (GPC), nuclear magnetic resonance (NMR), AFM, etc. [19]. This paragraph briefly describes the principles and applications of several of these new detection methods and summarizes the advantages and shortcomings or limitations of FTIR, GPC, NMR, and other methods.

**Figure 1:**
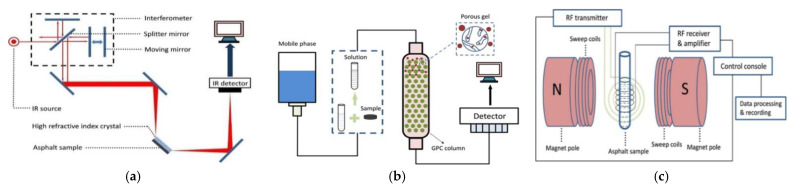
Schematic diagrams of three common chemical detection instruments: (**a**) FTIR, (**b**) GPC, (**c**) NMR.

FTIR has two modes of operation, projection and reflection, and because the reflection mode does not require passage through the asphalt sample, the reflection mode is more commonly used in asphalt inspection. The most commonly used reflection mode is the attenuated total reflection (ATR) mode. As shown in Figure 1a, the ATR-FTIR mode emits a beam of infrared light at a specific wavelength that is reflected by an interferometer and then directed at the asphalt sample, after which the infrared absorption spectrum of the asphalt sample is obtained [19,20]. FTIR can infer the type and content of the internal functional groups from the infrared spectrum of the asphalt sample, and then analyze the properties of the asphalt sample such as aging and regeneration [20,21,22]. In addition, it has been observed that the petroleum asphalt functional group is mainly C–H, whereas the bio-oil contains only about 65% of the total hydrocarbon content, so there are a large number of other functional groups present in the bio-oil [22,23]. Therefore, FTIR is also useful in the study of the properties of bio-asphalt.

According to the GPC principle shown in Figure 1b, the asphalt sample is dissolved with a solvent such as tetrahydrofuran to form the mobile phase, and the gel permeation chromatography is obtained by adding the mobile phase sample to the stationary phase of a porous cross-linked polymer gel to achieve the separation of molecules of different molecular weight sizes. By analyzing the molecular weight distribution and apparent molecular weight of the asphalt samples, the physical and chemical properties of the asphalt sample [24,25,26] including the modified properties of the modified asphalt [27,28] can be evaluated. As per the NMR principle shown in Figure 1c, by applying a strong magnetic field to an asphalt sample, molecules or nuclei with nuclear magnetism in the asphalt sample absorb the energy difference between the two energy levels that jump from the lower to the higher energy state, resulting in a resonance spectrum [29,30]. NMR uses the interaction of the applied magnetic field with the nuclei within the sample to detect phenomena from molecular and colloidal to macroscopic length scales, and the phenomena observed by NMR allow for structural analysis [31,32,33] and sample aging assessment [34,35]. As asphalt consists of organic molecules, H-1 NMR and C-13 NMR are currently the most commonly used in asphalt detection [30]. However, there are more than these two forms of NMR when it comes to asphalt assessment. Some other forms of NMR are used when assessing how other substances may affect the properties of asphalt such as Xin et al. [27], who used P-31 NMR to determine the hydroxyl values of partially depolymerized lignin (PDL) and a PDL epoxy monomer, as PDL is not made up of organic molecules like asphalt, which also contains other elements within it.

Table 1 shows that the three chemical analysis methods have clear advantages and are widely used in asphalt research, but they all have some disadvantages or limitations. In comparison, AFM has a broader application in asphalt research and the advantages of AFM over the other three chemical analysis methods are more evident. Researchers have used AFM to test a number of properties of asphalt and have achieved many results. The following section describes the principles of AFM and its application in asphalt testing.

### 2.2. Brief Introduction of AFM and Its Function on Asphalt

AFM is a new type of high-resolution instrument, originally developed by Binning and Quate [38] as an imaging tool, which can provide a picture of the shape of the sample surface. AFM imagines the surface using the interaction between a force-sensitive probe tip and the sample. The cantilever ends in a nanoscale tip, and the repulsion between the tip atoms and the sample surface atoms is weak. The height of the micro-layer of the sample can be detected by using the extremely small tip at the end of the cantilever. In the scanning process, the change in the micro-surface height will change the gravitational or repulsive force between the tip and the sample surface [39]. The force can be obtained by measuring the microcantilever shape variable and is closely related to the distance. In the scanning process, the feedback loop is used to keep the force and shape variable between the tip and the sample unchanged. The surface topography of the sample can be obtained by recording the track of the up–down movement of the tip. This detection method is called the constant force mode, which is also the most widely used scanning method of AFM.

According to Hooke’s law, when the cantilever is subjected to a variable force between the tip and the specimen, the cantilever with the tip deflects and moves upward in the direction perpendicular to the surface of the specimen. Normally, this shift causes a different signal to be reflected from the laser source shining on the back of the cantilever. The motion of the cantilever beam makes the laser beam deviate from the original position, and the measured voltage is converted to each capillary force, and then the image can be obtained from the measured signal [40].

When the tip of the AFM interacts with atoms on the surface of a sample, several forces usually act simultaneously on the microcantilever, the most important of which is an intermolecular force called van der Waals forces [41]. The van der Waals forces between the tip and the sample surface vary with the distance between them [42]. As the atoms at the tip and the sample surface approach each other, they first attract each other, and as the distance between them continues to decrease, the repulsive forces between them will begin to cancel out the attractive forces until the forces are in equilibrium. The distance is smaller, and the repulsive force between the two is dominant. If the needle tip has a different spacing from the sample, the AFM can be in different working modes. According to the different structural characteristics of the sample surface, material characteristics, and different research needs, the appropriate working mode is selected. The specific working mode will be introduced in the next section.

In recent years, Aguiar Moya et al. [43] estimated the mechanical properties of asphalt such as adhesion, hardness, and elastic modulus by AFM with nano indentation technology. Zhang et al. [44] analyzed the microstructural changes of asphalt after ultraviolet aging and water aging by atomic force microscope. Ji et al. [45] studied the microstructure of different modified asphalts by AFM at room temperature and low temperature. Guo et al. [46] observed the microstructure of different proportions of short-term aging and long-term aging on fusion asphalt.

The microstructure of asphalt is still an immature subject. Since Loeber first used AFM to observe the microstructure of asphalt in 1996 [47], many researchers have carried out various studies on asphalt using AFM and obtained a lot of information related to the morphology of asphalt samples. In the application of AFM in asphalt, the requirements for the experimental environment are not very high, and the sample preparation process is relatively simple. However, different temperatures and different sample preparation methods lead to significant differences in the final test results.

## 3. AFM Test on Asphalt Material

AFM can obtain the parameters of different physical properties by measuring the force between the probe and the sample and analyzing them. This section reviews the history of the application of AFM in asphalt materials and summarizes different sample preparation methods and common AFM testing modes.

### 3.1. The History of Using AFM on Asphalt

In 1986, Binnig et al. [38] developed a new type of microscope named the atomic force microscope, which combined the principles of scanning tunneling microscopy and stylus profilometry in order to measure very small forces and obtain surface images on an atomic scale.

**Figure 2:**
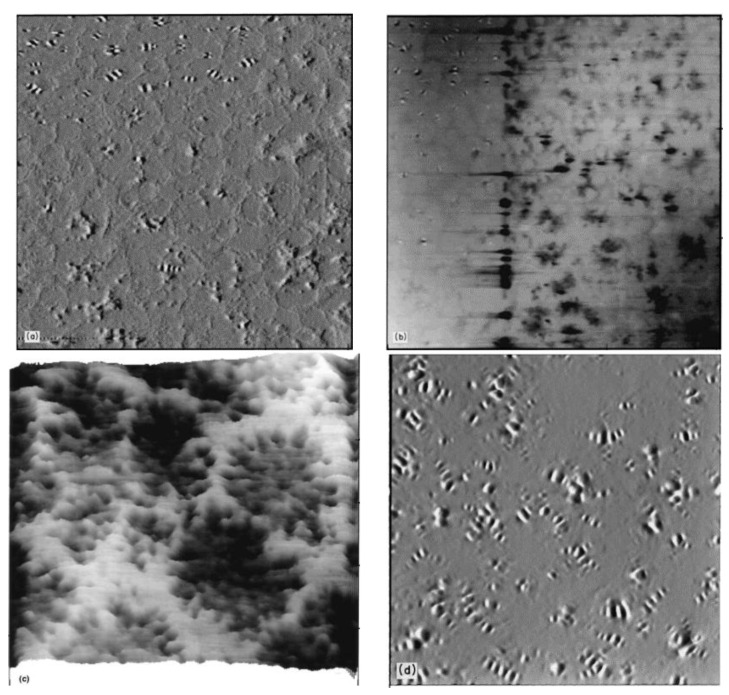
The microscopic images of asphalt surfaces. (**a**) AFM image of a gel asphalt taken by force mode. The bee-like structure is visible at the top. (**b**) AFM image of a gel asphalt taken by force mode. On the left side the bee-like structure is visible; on the right side the network structure appears. (**c**) AFM image of the same gel asphalt taken by the height mode after scanning at high magnification and presented in a 3D perspective view. (**d**) AFM tapping mode image of a gel asphalt. Reprinted with permission from [47]. © 2022 John Wiley and Sons-Books.

After years of research on asphalt, researchers have concluded that asphalt consists of four main components including a saturated fraction, aromatic fraction, asphaltene, and resin, which are often referred to as the ‘SARA’ components of asphalt. In 1996, Loeber et al. used AFM for the first time to examine asphalt without any pre-treatment, revealing the relationship between asphaltene molecules and asphalt gels. At the same time, their study revealed the existence of a wavy structure on the surface of asphalt, which they named the bee-like structure. According to Figure 2, Loeber et al. observed the appearance of bee-like structures at the top of the AFM images, and suggested that the appearance of bee-like structures was associated with asphaltene. In the study, the resolution of AFM was lower compared to that of instruments such as scanning electron microscope (SEM), but the AFM sample preparation method was simpler, which was one of the important reasons why AFM was later used by researchers for asphalt detection.

**Figure 3:**
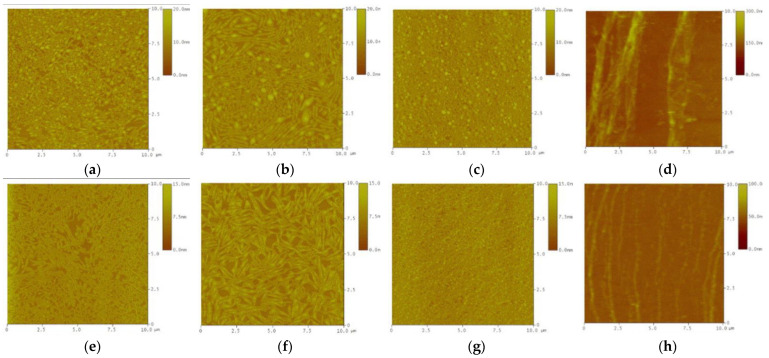
AFM images of monolayer LB films of high and low molecular weight asphaltene fractions deposited at different surface pressures π using the Langmuir-Blodgett method. (**a**) high-molecular-weight, π = 0 mN/m. (**b**) high-molecular-weight, π = 5 mN/m. (**c**) high-molecular-weight, π = 30 mN/m. (**d**) high-molecular-weight, π = 70 mN/m. (**e**) low-molecular-weight, π = 0 mN/m. (**f**) low-molecular-weight, π = 5 mN/m. (**g**) low-molecular-weight, π = 30 mN/m. (**h**) low-molecular-weight, π = 65 mN/m. Reprinted with permission from [50]. Copyright © 2022 Elsevier.

A key early technical tool used to observe the asphalt surface morphology was the use of the Langmuir–Blodgett technique, where asphalt samples were made into monolayers of Langmuir films. Ese et al. [48] used the Langmuir–Blodgett technique to transfer a monolayer of asphaltene and resin from the water surface onto a mica substrate, and subsequently used AFM to examine the morphology of these layers [49]. The results showed that a gradual increase in resin concentration resulted in the opening of the rigid asphaltene structure to a more resin-like structure. Zhang et al. [50,51,52,53] extracted and fractionated asphaltene from Athabasca oil sand asphalt using n-heptane, deposited the asphaltene onto silicon wafers using the Langmuir–Blodgett technique, and used the contact and tap modes of AFM to separately characterize the deposited LB (Langmuir–Blodgett) films. As per the images of the asphaltene LB films shown in Figure 3, high and low molecular weight asphaltene LB films exhibited similarities in behavior at the air–water interface under different pressures and revealed the presence of nanoscale aggregates in asphaltenes. Li et al. [54] also used AFM to characterize the asphaltene-deposited LB films and the results were similar to those of Zhang et al. where the asphaltene monolayers were more flexible at the heptanol–water interface than at the air–water interface.

Since Loeber and others [47] first examined asphalt using AFM and successfully observed bee structures, it has received a great deal of attention from researchers who believed that bee-like structures are strongly related to the numerous properties of asphalt. Jäger et al. [55] observed five different asphalt surface morphologies using AFM, successfully observed the bee structure, and observed hard bee, hard matrix, soft matrix, soft bee phases on the asphalt surface. Similar to the results of Loeber et al. [47], their study also showed that the presence of bee-like structures was associated with asphaltene. Masson et al. [56] first used low-temperature atomic force microscopy and phase detection microscopy to describe the nano- and microstructure of asphalt. The results were explained according to the glass transition temperature (Tgs) of the asphalt fraction. The domains visible under the microscope, the catena, peri, and para phases, are considered to be rich in asphalt, naphthenic, and polar aromatic hydrocarbons and saturates, respectively.

Force measurement is a traditional test in AFM. Traditional force measurement has many drawbacks including calibration, slow data evaluation, and low imaging resolution. Today’s force measurements have been greatly improved compared to traditional force measurements including rapid imaging and data evaluation as well as increased imaging resolution. Liu et al. [57,58,59,60,61,62] conducted numerous experiments using the force measurement mode of AFM in order to investigate the colloidal and surface forces between asphalt–silica particles, asphalt–asphalt, asphalt–fine powder, asphalt–silica, and asphalt–clay. They performed force measurements by generating an output signal as the sample plate approached the upper particle at the cantilever and quantifying the repulsive and attractive forces by an increase or decrease in the output signal. In addition, Abraham et al. [63] used the force measurement mode of AFM in asphalt testing to study the interaction between asphaltene and silica surfaces in aqueous solutions. Their study showed that the interaction between asphaltene and silica surfaces in aqueous solutions has a strong dependence on time, pH, and salt concentration. Drelich et al. [64] similarly measured the surface forces between silicon nitride AFM tips and Athabasca asphalt deposits in solutions of different pH and salt concentrations. They found that structural domains of very a small size existed on the asphalt surface and that the surface charge density of these structural domains varied stochastically. Long et al. [65] used AFM to measure the surface forces of individual hydrolyzed polyacrylamide (HPAM) molecules in desorption/adhesion forces on silica, mica, and asphalt surfaces. It was shown that there were differences in the adhesion forces of individual HPAM molecules to silica, mica, and asphalt, suggesting that HPAM would be beneficial in the recovery of asphalt from oil sand ores.

In later studies, AFM was used more often to study other aspects of asphalt properties. AFM is often used in conjunction with nanoindentation when studying the mechanical properties of asphalt to examine the stiffness properties, elasticity, and viscoelasticity of asphalt. Tarefder et al. [66] used AFM needles functionalized by different functional groups to examine their adhesion with dry and wet styrene-butadiene (SB) and styrene-butadiene-styrene (SBS) modified asphalt. The results showed that the adhesion results of the AFM probes with SB and SBS modified asphalt were different under different functional groups and different wet and dry conditions. Allen et al. [67,68] conducted their AFM with nanoindentation at its tip by improving it, based on which they conducted a series of studies on asphalt including its aging and viscoelastic properties. Nazzal et al. [69] used AFM tap patterns, force spectral analysis, and nanoindentation to study the adhesion of nano clay materials in the adhesion and cohesion of bituminous materials and summarized the principles explaining the mechanical behavior of bituminous clay nanocomposites. In addition, to reduce the effects of AFM tip contamination and operating parameters, a systematic procedure for measuring asphalt adhesion was developed by Yu et al. [70].

**Figure 4:**
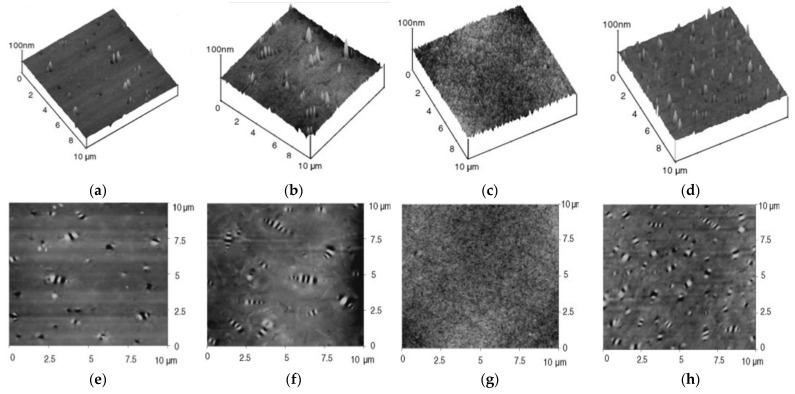
The three-dimensional and two-dimensional AFM images: (**a**) three-dimensional, unaged AH70; (**b**) three-dimensional, PAV aging AH70; (**c**) three-dimensional, unaged PG76; (**d**) three-dimensional, (**e**) two-dimensional, unaged AH70; (**f**) two-dimensional, PAV aging AH70; (**g**) two-dimensional, unaged PG76; (**h**) two-dimensional, PAV aging PG76; PAV: 1200 h, 60 °C and air pressure at 2.1 MPa. Reprinted with permission from [72]. Copyright © 2022 Elsevier.

The aging of asphalt is a very important characteristic of asphalt and leads to changes in the internal structure of asphalt as well as changes in the properties, resulting in a decrease in the asphalt road performance. AFM provides an opportunity to explain the aging of asphalt at a microscopic scale. Wu et al. [71] observed pure asphalt and ultraviolet (UV) aged asphalt by AFM and found an increase in the bee-like structure on the surface of the aged asphalt. The reason for this phenomenon may be due to the increase in asphaltenes with large molecular masses. The increase in asphalt mass was presumed to be due to the increase in aged asphalt. In later experiments, Wu et al. [72] also aged substrates and SBS modified asphalt using a rolling thin film oven (RTFO) and pressure aging vessel (PAV) and observed the changes in microstructure by AFM. As shown in Figure 4, after PAV aging, the surface of the asphalt became rougher, more tubers appeared, and there was an increase in bee structures, indicating that the asphalt produced more asphaltene micelles after PAV aging. Rebelo et al. [73] used AFM techniques to study the short- and long-term aging of asphalt cements and obtained similar results and found that the aging process was accompanied by a reduction in the malt phase. Menapace et al. [74] observed the micromorphology of both types of asphalt after short-term and long-term aging by AFM and found that aging had little effect on the micromorphology of warm mix asphalt (WMA), suggesting that the bee-like structure started from dendritic development. Allen et al. [67,75] observed the effect of oxidative aging on the microstructural properties in asphalt binders by AFM. The results showed that oxidative aging led to a large number of microstructural changes of different forms and degrees in different asphalts, and a striking correlation was found between the chemical parameters of the saturant and the effect of oxidative aging on the asphalt behavior.

**Figure 5:**
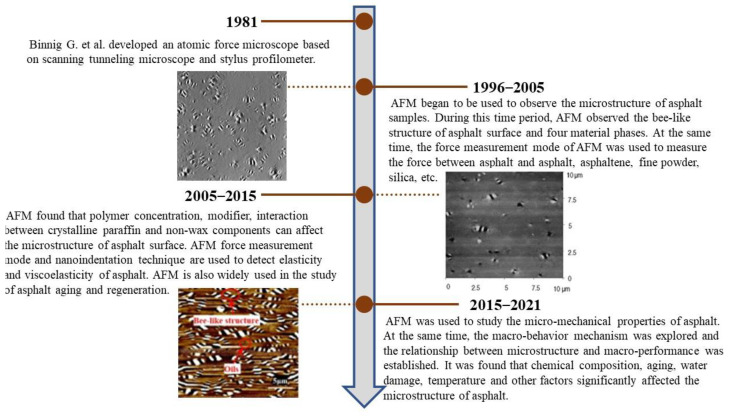
The history of the use of AFM to analyze asphalt [50,72,76]. © The Authors. Published by Elsevier.

Figure 5 shows the history of the use of AFM in asphalt inspection. From the history of the development of AFM in the field of asphalt testing, AFM was first heavily applied to observe the microstructure of the asphalt surface, while in this period, researchers used force measurement mode to measure the force between asphalt, fines, silica, mica, etc. As AFM equipment and techniques have developed and evolved, the use of AFM by researchers for asphalt testing has become more widespread, and AFM testing is increasingly being integrated with asphalt roadworthiness including asphalt aging and regeneration, and water damage to asphalt.

### 3.2. Sample Preparation Methods

AFM sample preparation is relatively simple compared with other observation methods. As shown in Table 2, hot casting method and solution casting method are the two most commonly used methods in the preparation of asphalt samples characterized by AFM. In order to obtain a smooth surface for AFM observation, the temperature of the sample preparation, the amount of asphalt, and whether the solvents involved vary from one researcher to another during sample preparation. At present, there is no official specification to use AFM to observe asphalt. Observations will vary due to inconsistencies in the preparation procedures including apparent differences in processing procedures and storage conditions. The surface roughness of thin films should be small enough to eliminate artifacts caused by uneven contact between the tip and sample when imaging.

In recent years, most researchers have used the hot casting method for experiments. The general process includes heating the binder in the oven, shearing and mixing the binder and asphalt, placing the asphalt on the glass slide, etc.

In order to ensure the smooth surface of asphalt, Yuan et al. [79] put a glass slide in an environment of 160 °C for 5 min, and further smeared the binder with a dagger. Aljarrah et al. [80] added the percentage of all modifiers according to the weight of the binder, and then placed the glass sample in an oven at 180 °C for 2 to 3 min until the surface was observed to be uniform and smooth. Israel et al. [81] hot-casted 15% CR-B samples from several millimeters of asphalt surface at room temperature with a laboratory knife to avoid surface oxidation. Li et al. [82] used glass rods to deposit molten asphalt on the glass slide to prepare AFM specimens, and then placed the droplets in an oven with a tilt of 30° above the horizontal direction, heated, and used gravity to coat a thin layer of asphalt on the slider surface.

It is worth noting that after preparing the samples, the researchers need to keep the samples sealed in Petri dishes or other containers from the completion of the sample preparation to the test period to prevent particles such as dust from gathering on the samples.

Another sample preparation method, the rotary casting method, focuses on casting the sample on a rotary plate. The centrifugal force of the plate is used to evenly spread the sample to form a film for imaging. When solvent casting is used, methods such as annealing the film at higher temperatures or freeze-drying the asphalt solution after deposition on the substrate are used to fix the sample, evaporate the solvent, and reduce molecular interactions. Most researchers have adopted the method of treatment at a higher temperature [45,83,84]. After thermal oxidation, asphalt is mixed with a certain amount of waste engine oil (WEO) or a stabilizer with sulfur as the main component. Then, it is rotated and sheared on a level table in oil bath or another high temperature environment. Finally, the asphalt is dropped on the glass slide by a glass rod or other equipment, and the asphalt is paved by heating the bottom to ensure the smooth surface of the sample. At the same time, it is fixed after cooling at room temperature.

As many researchers have used AFM to study asphalt samples, it is believed that specifications can be established to standardize the preparation process of hot cast samples including, but not limited to, temperature environment and binder type and other factors. At the same time, in order to better understand the influence of sample preparation and film thickness on the morphological development of asphalt, AFM should be used to characterize the same asphalt prepared by hot casting and solution casting with different film thicknesses to make the observed results more convincing.

### 3.3. Different AFM Testing Modes

It can be seen from the previous sections that with the improvement in AFM resolution and technology, the observation level is continuously improved, and the scope of the study gradually expanded. However, different AFM imaging methods have different effects on the mechanical properties and microstructure of the captured samples [85]. This section briefly introduces the working principle of several imaging models. Various imaging methods have different effects on the acquisition of the microstructure and performance research of samples, so it is necessary to find a suitable imaging method.

**Figure 6:**
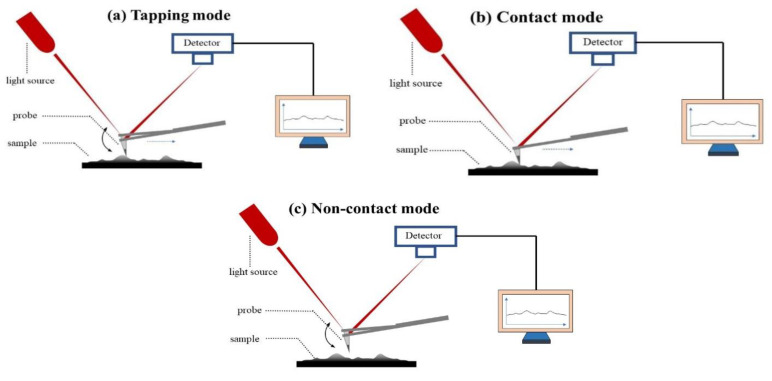
A schematic diagram of AFM with three different working modes: (**a**) tapping mode; (**b**) contact mode; (**c**) non-contact mode.

According to the force between the tip and the sample, the working modes of AFM are mainly classified into contact mode, non-contact mode, and tapping mode. As shown in Table 3 and the schematic diagrams in Figure 6, three different working modes are compared. The main differences are the distance between tip and sample and the moving direction of the probe. By analyzing various AFM imaging modes, the advantages and disadvantages of each mode are obtained, which is helpful in standardizing the application of atomic force microscopy in characterizing the microstructure of asphalt and evaluating the performance of asphalt.

#### 3.3.1. Contact Mode

The contact mode refers to the mode in which the tip maintains contact with the sample when the surface moves along the plane under constant load [40]. One end of the microcantilever, which is sensitive to weak force, is fixed, and the other end has a small tip. The tip is gently in contact with the sample surface [86]. During the contact process, the distance between the tip and the sample and the van der Waals forces generated are extremely small. There are two modes of imaging: constant force and constant height. The constant force mode refers to using the feedback system to accurately control the probe during the scanning process, so that it moves up and down vertically and horizontally with the surface morphology of the sample, and keeps a constant force between the tip and the sample, so that the deformation of the micro-cantilever remains unchanged [87,88]. In addition, the constant height mode is to keep the distance between the tip and the sample constant during the plane scanning of the tip, and the detector directly measures the deformation in the z direction of the microcantilever to form the topography image. Since the feedback loop is not used, the scanning speed of this method is fast, thus reducing the thermal drift effect. However, this method is not suitable for samples with large surface fluctuations [89,90].

#### 3.3.2. Tapping Mode

In tapping mode, the oscillating cantilever beam contacts the sample surface at the end of the swing, providing not only the height and amplitude images, but also the phase diagram. The phase contrast image is generated by recording the phase shift between the driving force and the tip response, so it contains the information of the mechanical properties of the sample [91]. Tapping mode is also regarded as intermittent contact mode. In this mode, the cantilever of the supporting tip oscillates near its resonant frequency, resulting in a tip oscillation range of 20–40 nm. The tip gently hits the sample surface, usually 200–400 times at each scanning point, and intermittently contacts the sample without contacting the surface, and moves in the X-Y direction [92]. This mode avoids the existence of lateral force, which can scratch or even damage the soft sample surface, reducing the probability of surface damage caused by shear stress, and has no effect on some samples that are not firmly bonded to the matrix. As a result, it is more suitable for imaging soft samples such as biomolecules and polymers than the contact mode.

#### 3.3.3. Noncontact Mode

In non-contact mode, the tip vibrates above the sample surface and never touches the sample, causing little or no damage to the sample surface [93]. The needle detector detects van der Waals and static forces and does not damage the imaging sample over long distances. In the non-contact mode, the cantilever maintains its natural frequency vibration, with the amplitude varying with distance from the surface.

Noncontact testing mode contains two main imaging modes: constant frequency offset and constant height. In the constant frequency offset imaging mode, the tuning fork cantilever is kept at a constant amplitude through the amplitude feedback loop, and the distance between the tip and the sample is adjusted through the frequency feedback loop to keep the frequency offset constant [94]. The obtained image is the height map of the sample surface morphology at the constant force gradient. In constant height imaging mode, the feedback loop of the frequency offset control is disconnected to keep the tip height constant, and changes in frequency offset during scanning are detected.

Although this mode increases the sensitivity of the microscope, the resolution is lower and the scanning speed is slower than the contact and tap modes because of the larger distance between the tip and the sample surface and the measurement in the change in the natural resonance amplitude of the cantilever [40]. In addition, this mode is relatively difficult to operate and is usually not suitable for imaging in liquid. It can only be used for samples with a hydrophobic surface, so it is rarely used.

## 4. AFM Data Analysis

This section summarizes the latest research results of AFM on polymer modified asphalt and asphalt aging and regeneration. The analysis of the data allows for the common conclusions of experts and scholars and the still controversial areas to be drawn for further development in the future.

### 4.1. Effect of Polymer Modification

Asphalt is composed of hydrocarbons with various molecular weights and non-metallic derivatives, which is used as a binder for aggregate. However, further applications of asphalt are limited due to high temperature rutting and low temperature cracking. To improve the quality of asphalt, it is usually modified by various polymers such as styrene butadiene styrene (SBS), waste crumb rubber (CR), styrene rubber (SBR), and ethylene acetate (EVA). In recent years, some experts and scholars have used AFM to analyze the microstructure of modified asphalt and analyze the relationship between its morphology and performance.

#### 4.1.1. SBS Modified Asphalt

SBS modified asphalt is able to simultaneously improve the low and high temperature performance of asphalt, making it the most studied and applied variety. Therefore, it is necessary to study the modification mechanism and the influence of its microstructure and morphology on the asphalt properties.

**Figure 7:**
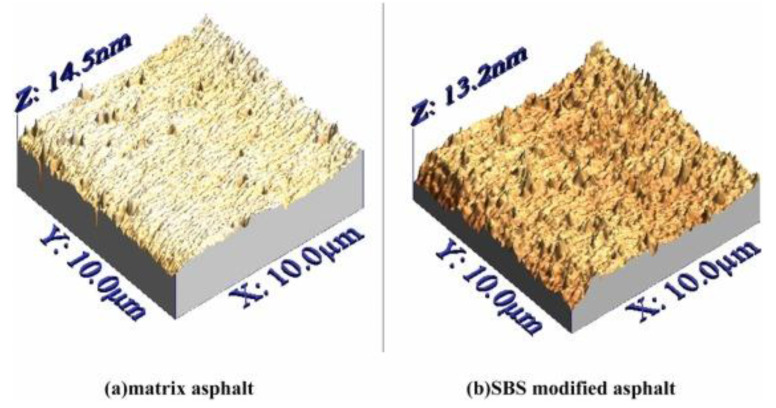
The bee phases of the matrix asphalt and SBS modified asphalt. Reproduced with permission of [93]. © 2022 Elsevier Ltd.

As shown in Figure 7, the addition of SBS increased the bee phase of asphalt and produced asphalt with better physical properties [93]. With regard to the adhesion characteristics of the SBS modified asphalt, the researchers found that the peak area ratio was considered to be the micro-viscosity characteristic of the polyphosphate (PPA)/SBS modified asphalt, which had the highest correlation coefficient with the macro adhesion performance index. The bonding performance and peak area ratio of the modified asphalt decreased with increasing PPA and dibutylphthalate (DBP) content, while the bonding performance increased and then decreased with increasing SBS content [95].

**Figure 8:**
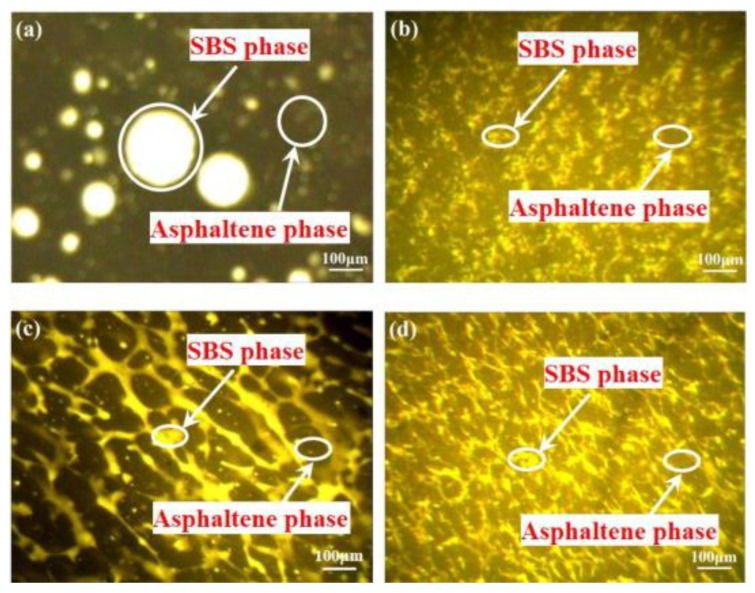
Fluorescence micrographs of (**a**) SBS–modified asphalt; GO/SBS-modified asphalt with different GO content of (**b**) 0.1 wt%; (**c**) 0.3 wt%; (**d**) 0.6 wt%. Reproduced with permission of [76]. © 2022 The Authors. Published by Elsevier Ltd.

There have been many studies on the bonding system of modified asphalt in the microstructure. Adding graphene and other materials can improve the bonding performance and other physical properties of asphalt. Some researchers have found that nanocells improved the moisture resistance and bonding strength between the polymer and the modified adhesive system [96]. The residual bee structure in the nanocell modified asphalt was higher than that in the SBS modified asphalt, indicating that the addition of nanocells made the surface morphology of asphalt more resistant to water damage [96]. In addition, the addition of nanocells could also increase the roughness of the SBS modified asphalt. The researchers also found that SBS modified asphalt with 0.3% graphene oxide showed good viscoelasticity, and the analysis showed that the modified asphalt had a higher rutting factor and lower damping factor. As shown in Figure 8, 0.3 wt% GO/SBS modified asphalt had the most developed polymer-rich phase, while SBS modified asphalt was the least developed, which shows that graphene oxide promotes the development of a polymer-rich phase in modified asphalt, and a continuous polymer-rich phase indicates good pavement performance [76].

In addition, it was found that adding waste engine oil bottom (WEOB) could improve the colloid content and polarity of the SBS modified asphalt and make the polymer structure dense. From a microscopic point of view, WEOB promotes the polarity of SBS and forms the grafted product MAH-g-SBS with asphalt, which inhibits the thermal movement of molecules and improves the high temperature rutting resistance or elasticity, and the low temperature fatigue cracking resistance of asphalt. WEOB can also promote the development of a SBS network structure and improve the swelling properties and compatibility of asphalt [97,98,99].

For the other physical properties of SBS modified asphalt, it was found that the permeability decreased and the softening point increased as the area of the bee structure of the SBS modified asphalt increased. In addition, the SBS content of 4% is a critical point. When the SBS content was less than this critical point, the bee structure area increased gradually and the roughness decreased, but the trend was not obvious when the SBS content exceeded this critical point [100].

In the last five years, SBS modified asphalt has been studied by AFM, and the AFM images are related to the adhesion performance index or adhesion strength and roughness of the asphalt. Through the research and comparison of different experts and scholars, it was found that the addition of nanocells, graphene oxide, WEOB, and other substances was conducive to the formation of a better bee structure and improved the low temperature and high temperature performance of the SBS modified asphalt.

#### 4.1.2. CR Modified Asphalt

AFM is a new and effective tool for the further observation and analysis of microstructures. With the rapid development of the transportation industry, the intensity of the vehicle axle load has increased, and the performance requirements of asphalt pavement are gradually improving.

Crumb rubber belongs to rubber, which has a certain bonding ability with asphalt. Waste crumb rubber can be used in asphalt pavement construction to develop environmentally friendly and sustainable pavements. When added to asphalt, it can improve road fatigue and rutting resistance. However, due to the large amount of crumb rubber, the stability of modified asphalt is not good, and it is easy to produce stratification and segregation, which affects its performance. Using AFM to observe the microstructure of modified asphalt is helpful to further study and improve the performance of asphalt.

**Figure 9:**
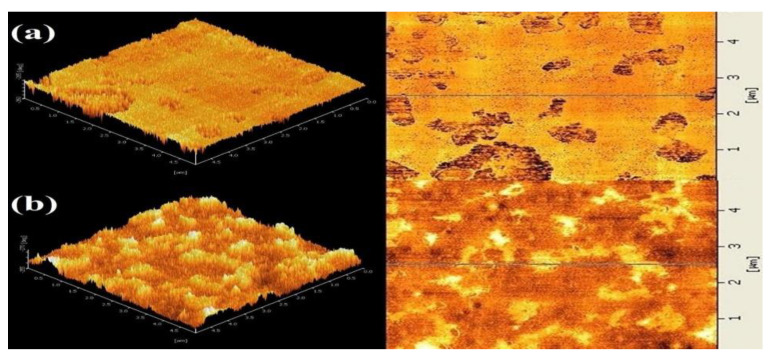
Phase image. (**a**) Neat binder; (**b**) CR modified binder. Reprinted with permission from [102]. Copyright © 2022 Elsevier Ltd.

Some experts and scholars have found that the viscosity of the binder will increase after adding CR. Microscopic manifestations are centered on bee-shaped structures, which increase in number and decrease in size, leading to improved durability of the road [101]. Researchers have also studied the rheological, morphological, and physical changes of asphalt binders with different CR content using wet interaction. It was found that under AFM tapping mode, the softening point and viscosity increased with the increase in CR content, while the permeability and ductility changed in the opposite manner. In the process of mixing and interaction, the most important influencing factors are temperature and time. The phase images of the neat binder and CR modified binder are shown in Figure 9, where the darker quasi-phase region increased due to the swelling effect absorbed by the CR particles after adding the rubber powder binder. It was found that the interaction between the rubber and the neat hopper was optimal when 10% CR was added in the range of 170–180 °C, resulting in a higher hardness and elasticity of the rubberized asphalt, thus improving its resistance to permanent deformation [102]. In addition, as the modification time increased, the chemical changes in the CR modified asphalt mainly occurred in the quasi-structure, and the bee structure decreased [81]. Therefore, for the design of a suitable polymer modified asphalt, it is necessary to consider the specific conditions of each case including the action temperature, transportation, and construction time.

According to the different types of CR, the adjustment time has a remarkable influence on the performance of the asphalt mixture. The AFM-IR results under different time conditions showed that after a long treatment time, the microstructure bee structure developed poorly, and the main chemical changes of functional groups occurred in the para-domain.

For the modified asphalt mixture of CR and SBS, some experts and scholars have studied the influence of their composition and rheological properties on the modification mechanism. It was found that the free radicals of CR interact with the amide groups in WEOB to form a semi-continuous phase [99].

In addition, the surface roughness is also an important standard to measure the physical properties of asphalt. The data obtained from the tests can also be related to the macroscopic bonding performance of asphalt. The lower the roughness, the smaller the bonding force. For asphalt without a bee structure, the surface energy of the material can be used to reflect the physical bonding properties of the material. The higher the surface energy, the stronger the adhesion between the asphalt and rubber.

There is a relationship between the surface roughness and adhesion properties of bee structures. Researchers have found that the surface roughness of asphalt samples increased with the addition of silicone oil, indicating that silicone oil contributed to the binding of asphalt and CR as well as the continuous and uniform dispersion of rubber particles in asphalt [103].

It can be seen from the above that the addition of different binders or other substances can improve the surface roughness and bonding properties of asphalt, which is conducive to the integration of CR and ordinary asphalt, thus improving the stiffness and elasticity of modified asphalt materials, and further improving the rutting resistance of roads.

#### 4.1.3. SBR Modified Asphalt

Styrene butadiene rubber (SBR) is the world’s most widely produced and consumed common synthetic rubber, which is prepared by free radical emulsion polymerization or anionic solution polymerization with butadiene and styrene as monomers. SBR modified asphalt prepared by adding SBR as a modifier to asphalt has excellent low temperature ductility and cracking resistance, which is suitable for use in cold regions like other additives such as WHA [104,105,106].

In addition to improving the cold resistance, experts and scholars have also improved the hot mix asphalt performance of SBR modified asphalt by adding other modifiers. Some researchers have studied the performance characteristics of a nano-CaCO_3_/styrene-butadiene rubber modified asphalt mixture, and the styrene-butadiene rubber modified asphalt was mixed with nano-CaCO_3_ particles according to the different contents of asphalt binder. The rutting test was used to determine the content of the nano-composite modifier, which had the same high temperature deformation performance as SBS modified asphalt mixture. The microstructure and micro-mechanical properties of the modified asphalt were evaluated by AFM, and the modification mechanism of the nano-modifier was studied. The experimental results showed that 5% of the CaCO_3_/SBR modifier could be well-dispersed in asphalt, which significantly improved the micro-mechanical properties such as the adhesion and dissipation energy of asphalt, but had a negative impact on the modulus of Derjaguin–Muller–Topolov (DMT) [107]. This indicates that it has good potential for the comprehensive improvement in the hot mix asphalt performance, which is expected to be applied in warm regions.

**Figure 10:**
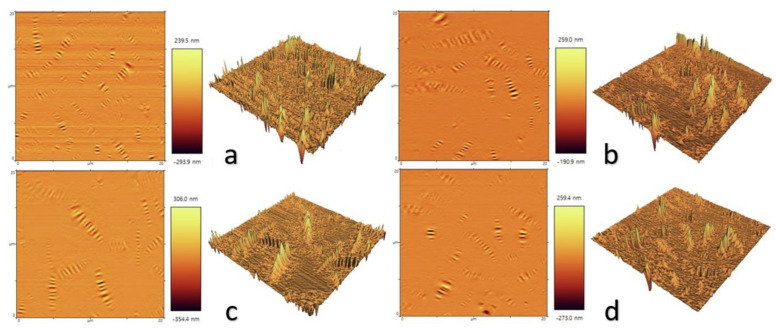
The topographic images of the unmodified and modified binders. (**a**) Control; (**b**) Soap; (**c**) Post; (**d**) Co-mill. Reprinted with permission from [108]. © 2022 Elsevier Ltd.

For further study on improving the durability of SBR modified asphalt, researchers have used the soap pre-batch method, post-mixing method, and co-grinding method, and added SBR into the emulsion to study the effect of the preparation method of the polymer modified asphalt emulsion on the microstructure and mechanical properties of cold recycled mixtures [108]. Figure 10 indicates that the distribution of the bee structure on the topographic map is not uniform, and the nanoscale asphalt is a composite material composed of different phases rather than a completely uniform material. Through AFM testing and analysis, a significant relationship was found between the elastic modulus of the asphalt and the dynamic modulus of the asphalt mixture. The AFM surface roughness of the asphalt was inversely proportional to its adhesion, and samples with higher adhesion in the AFM test were more durable in the mechanical tests.

### 4.2. Effect of Aging and Rejuvenating

Asphalt aging refers to the effect of environmental factors and vehicle load on asphalt, which will lead to a series of changes such as volatilization, oxidation, and polymerization, resulting in changes in the internal structure and performance of asphalt [109]. Asphalt aging is characterized by surface drying and embrittlement, followed by cracking and a loose surface. In terms of technical indicators, the asphalt viscosity and softening point increase, and the penetration and ductility decrease [102]. The aging of asphalt weakens the binding force between the asphalt and aggregate particles, leading to the hardening of the asphalt concrete pavement, the loose shedding of pavement particles, and the reduction in road durability. Asphalt regeneration is the inverse process of asphalt aging.

In order to reduce the influence of aging on asphalt morphology, researchers have studied the microstructure level of the original binder containing crude palm oil and the recycled asphalt pavement (RAP) binder and found that the stability and indirect tensile strength of the RAP materials increased to 80% with the increase in the amount of RAP materials [110]. Research has shown that the addition of crude palm oil (CPO) as a reactivation agent increased the heterogeneity of the morphology. Due to the effect of aging on the binder, it was observed that the bee structure became thinner, the asphaltene content increased, and the corresponding resin content decreased. Other experts and scholars have analyzed the aging index and surface morphology of the clay asphalt of limestone and fly ash where the aging index of the asphalt adsorption sample of fly ash was lower than that of the corresponding limestone mortar sample. When the asphalt adsorption sample was close to the surface of the limestone particles, the number of lamellar structures increased, and the size increased first, and then decreased. Therefore, the interaction between particles can improve the anti-aging performance [111].

**Figure 11:**
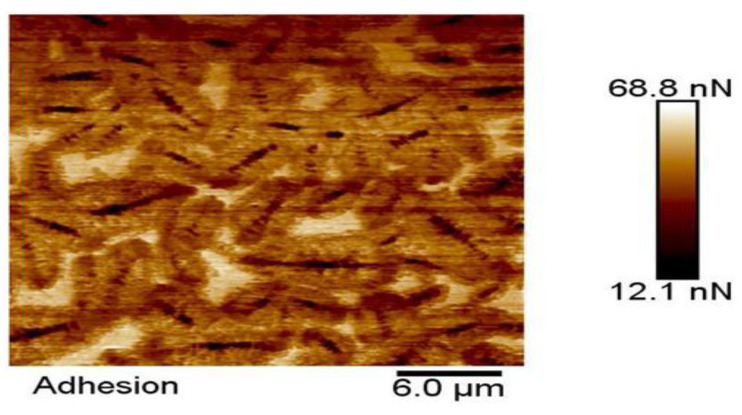
Micro-adhesion diagram of the asphalt binder using the DMT mode in AFM. Reprinted with permission from [76]. © 2022 The Authors. Published by Elsevier Ltd.

Regarding the aging of modified asphalt, different experts and scholars have used different indices in recent years including, but not limited to, the colloidal index, creep stiffness, surface roughness, etc. to study the asphalt properties. Obvious asphaltene aggregation occurs during asphalt aging, and the higher the resin index, the easier the asphaltene can be dissolved in an oil-based medium. The dispersion domain containing asphaltene is easily dissolved by other components in the original asphalt sample, resulting in little difference between the matrix and dispersion domain [112]. As shown in Figure 11, the researchers found that the bee structure of the SBS modified asphalt increased after aging, and the adhesive showed a point structure. The aromatic oil had a close recovery effect on the microstructure of the SBS modified asphalt [76]. In addition, adding bio-oil to asphalt can reduce the formation of ester-related new peaks and aromatics, and adding a bio-adhesive can reduce the saturated hydrocarbons, aromatics, and asphaltenes, and increase resin [29]. The molecular weight and molecular kinetic energy of the aged and modified asphalt increased, and the radial distribution function (RDF) peaks between the asphaltenes decreased. The asphaltenes were less likely to aggregate, were more dispersed in the molecular model, and had a uniform surface microstructure.

The addition of graphene, ethylene copolymer binder (ECB), and other substances also has a corresponding impact on the aging of the modified asphalt. For the microscopic morphology study of graphene-modified asphalt aging, the researchers found that graphene could act as an additional dispersed nucleation center to promote the formation of many smaller bee structures, and can reduce the microscopic morphological changes after aging. Alkanes are arranged during the asphalt cooling process to form the nuclear embryo of the bee structure that promotes the nucleation and growth. It was found that the root mean square roughness (Rq) of the asphalt modified with graphene was lower than that of the base asphalt, indicating that graphene is helpful in improving the anti-aging performance [82]. The overall roughness of the asphalt binder increased with the increase in the aging level, and there was no linear relationship between the surface roughness and aging level [113]. Researchers have also found that the addition of ECB can improve the complex modulus of rubber asphalt binder, reduce its shear stress sensitivity, and improve the aging resistance [114]. In addition, some experts and scholars have found that PAV aging reduced the number of bee structures and the surface roughness of the asphalt binder, and a rejuvenation agent increased the morphological fluctuation and surface roughness [115].

In addition to conventional performance indicators, asphalt materials are affected by environmental factors in the use process, leading to asphalt aging and reducing the asphalt pavement performance. Temperature is the most important factor affecting asphalt aging in the environment, but has not well-studied yet. In recent years, researchers have found that with the increase in dynamic thermal aging cycles, the contents of saturated hydrocarbons and aromatic hydrocarbons in asphalt decreased, and the asphaltene content increased significantly [116]. The aged asphalt gradually became hard and brittle, especially after the dynamic peak temperature was 200 °C. Through dynamic thermal aging, the roughness and height of the bee structures decreased [117]. The researchers also found that with the increase in the aging degree, the ductility of the asphalt decreased, but the critical temperature at low temperature increased. The bee structures between the light and dark phases on the asphalt surface decreased with the increase in the aging degree, but the distribution was more dispersed [44].

In summary, experts and scholars have used AFM to observe the asphalt before and after aging. Combined with the morphology and quantity of the bee structures and the analysis of various physical properties, it was found that the number of asphalt aging bee colony structures decreased and the distribution was more dispersed. In the chemical composition, the contents of saturated aromatic hydrocarbons decreased and the asphaltene content increased. The addition of RAP, CPO, ECB, PAV, and other substances can improve the anti-aging performance of asphalt and slow down the aging of asphalt. Moreover, the change in temperature will also reduce the number of bee structures and accelerate the aging of asphalt.

## 5. Future Development of AFM Testing

The surface of asphalt has different microstructures, among which the bee structure is mainly studied by AFM. The microstructure of asphalt has different degrees of development according to its original source and the sample preparation methods. In the past thirty years, experts and scholars have continued their efforts on the origin of typical bee structures.

It is difficult to distinguish between different types of asphalt from the appearance of asphalt. Observing the microstructure of asphalt with AFM can help to determine the type of asphalt accurately. However, AFM mainly studies the microstructure of the asphalt surface, and asphalt itself has the characteristics of non-uniformity and easy cracking. It is doubtful whether it can represent the molecular arrangement in a large volume of asphalt blocks.

There are still some problems to be solved in the chemical composition and mechanical connection of asphalt. Asphalt has complex chemical properties, and AFM has certain limitations in chemical composition characterization, so it is still unable to distinguish a specific asphalt chemical composition according to different morphological domains of the microstructure. In the future, combining AFM with other chemical analysis tools to study and analyze the same asphalt will also provide a way to connect the chemical composition with the microstructure of the asphalt.

In the future, it is necessary to address the technical challenges related to the AFM characterization of asphalt materials such as the sample preparation, equipment observation level, AFM tip pollution contamination, data acquisition, and image analysis. At present, there is no complete standard to regulate the test behavior of the experts and scholars in the preparation of asphalt samples observed by AFM. The main reason is that the sample preparation methods are relatively diverse, and there is a lack of sufficient experimental data to justify a sample preparation method, especially the temperature conditions and sample preparation time limitations, which need to be supported by more experimental data.

In recent years, more experts and scholars have observed the bee structure in different asphalt samples and linked the bee structure to the physical properties of asphalt. They found that it is related to the performance indices such as the aging degree and anti-aging ability of asphalt. Researchers also expect to add binders, polymers, and other substances. However, there have been few studies on the simultaneous addition of multiple substances to asphalt mixtures, and there is no consensus on the influence of the addition of some additives to the performance of asphalt.

Therefore, the application of AFM in asphalt has a great development space in the future. There is a need to further explore the potential of AFM in characterizing the micromechanical properties of materials and to explore the relationship between the microstructure and material properties. The establishment and analysis of intelligent models will help to accelerate the application of AFM in asphalt and other industrial products and contribute to the further improvement of asphalt recognition and performance.

## 6. Conclusions

It has been demonstrated that AFM is an effective method to study both the morphology and micro-mechanics of asphalt polymers. It is able to predict the overall mechanical properties of asphalt and provide a scientific reference to analyze the mechanical behavior. The main objective of this paper was to review the state-of-the-art using AFM as advanced technology to better analyze the micro-mechanical behavior of various asphalt binders. The main conclusions are as follow:

1. AFM can be used to analyze the microstructure of asphalt binders at the microscale, which provides a new idea for the application of AFM in the field of asphalt materials and a further basis for the study of the asphalt microstructure. The “bee structure” of asphalt under AFM can represent the four components of asphalt (asphaltene, resin, saturate, and aromatic). The adhesion force among the four components in a single group can represent the force between colloid structures.
2. AFM can be used to investigate binder modification. It has been successfully used to acquire micro-mechanical information such as the relative stiffness/Young’s modulus, stickiness/adhesion, hardness, energy loss, and sample deformation quantitatively. Furthermore, the microscale changes correlated to the physical, chemical, and rheological performance of the modified binder.
3. AFM can be used to characterize the effect of short-term, long-term aging and UV radiation on the surface morphology and micro-mechanical properties of the asphalt binder. In addition, the blending between RAP and virgin binder, the devulcanization of the rubber in the asphalt binder, the healing characteristics, and the stress concentration due to phase separation can be studied by the “bee structures” and micro-mechanical behavior.

In terms of future challenges and opportunities, some recommendations on the development of asphalt AFM testing can also be given for future studies as follows:

4. The sample extraction and preparation to conduct AFM are very important factors. Standards or specifications are needed to obtain homogenous samples with a sufficient thickness and no surface contamination.
5. The combination of adhesion measurements with new AFM testing techniques such as localization atomic force microscopy [118] and fluidic force microscopy [119,120] may contribute to better characterization of the chemical and mechanical relationship of asphalt binders.

## Figures and Tables

**Table 1:** A summary of the advantages and disadvantages of FTIR, GPC, and NMR.

Chemical Detection Method	History	Testing Forms	Application in Asphalt Testing	Advantages	Inadequacies or Limitations
FTIR	1950s	Speculation of the type and content of functional groups in a sample from the absorption spectrum of an asphalt sample [24]	- Investigate the aging and regeneration properties of asphalt [20,25,26,28,29] - Evaluate the performance of bio-asphalt [22,23]	- Ability to display the type and content of functional groups in the sample - Low sample size required - Simple and fast experiment	- Sample preparation is cumbersome (especially in transmission mode) - Accuracy and resolution are not yet high
GPC	1960s	Separation of molecules of different sizes by passing the sample through the stationary phase of a porous cross-linked polymer gel with a mobile phase	- Evaluate the physical and chemical properties of asphalt [21,22] - Evaluate the modified performance of modified asphalt binders [22,30,31]	- Ability to characterize the molecular weight distribution and apparent molecular weight of a sample - Allow assessment of the physicochemical properties of asphalt	- There is a limit to the volume of the GPC drench, and the chromatography is only as good as the volume of the macromolecular compound within a specific mentioned range
NMR	1950s	Application of magnetic field interactions with nuclei within a sample to detect the length of phenomena from molecular and colloidal to macroscopic scales	- Analysis of the structure of asphalt [24,32,33] - Evaluate the aging properties of asphalt [34,36]	- Detailed information on the structure, chemical environment and functional groups of the asphalt binder can be reflected by the peak positions of the NMR [35] - NMR data combined with multiple regression methods for estimating petroleum properties	- C-13: high cost of analysis, large number of samples required - H-1: no signal can be obtained in relation to internal carbon, which is not directly bound to any hydrogen [35]
AFM	1980s	Detection of the weak interatomic interaction between the surface of the sample and the micro-force sensitive element [37]	- Characterization of microscopic morphology - Evaluation of aging regeneration properties - Measurement of forces	- Low environmental requirements and simple sample preparation process - Be able to provide three-dimensional surface map and observe non-conductive samples - Be able to work well under normal pressure even in liquid environment	- It has a small imaging range, slow imaging speed, and is greatly influenced by the probe.

**Table 2:** The difference between the two AFM sample preparation methods [56,77,78].

Method	Hot Casting	Solution Casting
Description	Drop a string of asphalt binder onto the substrate and heat it on the hot plate to convert the asphalt binder into liquid and spread it out with a blade to form a film.	The asphalt binder is dissolved in a specific concentration of organic solvent, and part of it is deposited on the glass slide in the centrifuge to obtain the asphalt binder film.
Thickness of film	Micrometers, depending on the amount of asphalt binders and substrate surface area.	Varies from nanometers to micrometers, depending on the rotation speed and the concentration of the asphalt binder solution.
Advantage	The solid morphology is preserved during experiment.	Great flatness of the surface.
Disadvantage	Heat treatment is required and the surface has lower flatness.	Because the solvent and evaporation process change the molecular interaction in asphalt binder, it has a greater impact on the microstructure.

**Table 3:** Differences between the three working modes of AFM.

Mode	Tapping Mode	Contact Mode	Non-Contact Mode
Advantage	Not affected by transverse force, reducing the force caused by the adsorption liquid layer, the image resolution is high.	Fast scanning speed	No force applied to the sample surface and no effect on the sample.
Application	Fragile or soft adhesive sample.	Hard samples with obvious changes in vertical direction.	Sample for hydrophobic surfaces.
Disadvantage	Scanning speed is lower than the contact mode.	The effect of lateral force and adhesion reduces the spatial resolution of the image, and the soft sample will be damaged when the tip scratches the sample.	The separation of tip and sample results in low lateral resolution and slowest scanning speed.

## Data Availability

The data presented in this study are available on request from the corresponding author.
